# Role of Fibre in Nutritional Management of Pancreatic Diseases

**DOI:** 10.3390/nu11092219

**Published:** 2019-09-14

**Authors:** Emanuela Ribichini, Serena Stigliano, Sara Rossi, Piera Zaccari, Maria Carlotta Sacchi, Giovanni Bruno, Danilo Badiali, Carola Severi

**Affiliations:** 1 Department of Translational and Precision Medicine, Sapienza University of Rome, 00161 Rome, Italy; emanuela.ribichini@uniroma1.it (E.R.); piera.zaccari@uniroma1.it (P.Z.); mariacarlotta.sacchi@uniroma1.it (M.C.S.); 2 Department of Internal Medicine and Medical Specialties, Gastroenterology Unit, Sapienza University of Rome, 00161 Rome, Italy; serena.stigliano@uniroma1.it (S.S.); dt.sara.rossi@gmail.com (S.R.); giovanni.bruno@uniroma1.it (G.B.); danilo.badiali@uniroma1.it (D.B.)

**Keywords:** pancreatitis, pancreatic exocrine insufficiency, nutritional management, maldigestion, diet, fibre

## Abstract

The role of fibre intake in the management of patients with pancreatic disease is still controversial. In acute pancreatitis, a prebiotic enriched diet is associated with low rates of pancreatic necrosis infection, hospital stay, systemic inflammatory response syndrome and multiorgan failure. This protective effect seems to be connected with the ability of fibre to stabilise the disturbed intestinal barrier homeostasis and to reduce the infection rate. On the other hand, in patients with exocrine pancreatic insufficiency, a high content fibre diet is associated with an increased wet fecal weight and fecal fat excretion because of the fibre inhibition of pancreatic enzymes. The mechanism by which dietary fibre reduces the pancreatic enzyme activity is still not clear. It seems likely that pancreatic enzymes are absorbed on the fibre surface or entrapped in pectin, a gel-like substance, and are likely inactivated by anti-nutrient compounds present in some foods. The aim of the present review is to highlight the current knowledge on the role of fibre in the nutritional management of patients with pancreatic disorders.

## 1. Introduction

### 1.1. Fibres in Human Health

According to major food and health organisations such as the European Food Safety Authority (EFSA), the Food and Agricultural Organisation (FAO)/World Health Organisation (WHO), the Scientific Advisory Committee on Nutrition (SACN), the Nordic Nutrient Recommendations (NNR), and the German Nutrition Society (DGE), people’s health benefits greatly from dietary fibre, e.g., on gastrointestinal disorders and risk in non-communicable diseases [1]. In regards to the most common diseases worldwide, several studies focused their attention on the protective effect of dietary fibre on cardiovascular disease (CVD) likely related to the reduction of metabolic syndrome (MetS), one of its promoting factors [2,3,4]. An inverse relationship has been found between high consumption of dietary fibre and MetS with a lower risk of MetS for every 10 g/day fibre increment, especially with cereal and fruit rather than vegetables [5]. Their positive effects on MetS are probably due to promotion of satiety, delayed gastric emptying, reduction of macronutrients absorption with subsequent weight loss in obese patients, improvement of insulin sensitivity, hypertension, reduction of cholesterol plasma levels, triglycerides levels and triglycerides to HDL-cholesterol ratio [4,6]. Similar studies in the pancreatic setting are conflicting. Recently, dietary fibre intake has been reported to be protective against the occurrence of acute pancreatitis (AP) by Setiawan et al. [7]. This is in contrast to a previous prospective cohort study [8] in which no association was found. Studies on the effects of dietary fibre intake on pancreatitis management are scarce and are hindered by the lack of specific pancreatic “disease indicators” as well as by the dichotomy of fibre effects, as discussed later, between pancreatic conditions with or without pancreatic exocrine insufficiency (PEI).

The main beneficial effects on human health are due to physicochemical fibre properties, namely their solubility in water, fermentability and viscosity. As far as solubility is concerned, soluble fibre dissolves in water and gastrointestinal fluids, is transformed into a gel-like substance, which is digested by bacteria in the large bowel, and gases and few calories are released. On the contrary, insoluble fibre does not dissolve in water or gastrointestinal fluids, it remains unchanged as it moves through the digestive tract, and therefore, does not result in a source of calories [9]. A healthy diet contains a mix of both soluble and insoluble fibre. Soluble fibre is more present in fruits (apple, citrus), vegetables, legumes, potatoes, seaweed extracts, microbial gums; while insoluble fibre is more common in plants (vegetables cell walls, sugar beet, various brans), green bananas and cereal grains [9,10]. 

Fermentability by bacteria in the large bowel contributes to microbial diversity, whereas viscosity, that is the capability of fibre to form a gel-like substance when dissolved in intestinal fluids, influences nutrients absorption, a property exploited to control glucose and lipid metabolism [9]. Besides the physicochemical properties of fibre, it also provides humans with vitamins, minerals and anti-oxidant compounds that can contribute to their health [11,12]. In adults, the recommended amount of dietary fibre ranges from 25 to 38 g/day, a high fibre diet being defined as fibre content higher than 25 g/day in women and 38 g/day in men [13].

The influence of dietary fibre rich food on gastrointestinal functions has been increasingly recognised [14,15,16]. Dietary fibre has been demonstrated to have a significant effect on fecal weight, intestinal motility and transit time. The increase in fecal volume and weight is due to the ability of fibre to absorb water and convey it into the bowel [17,18]. The consumption of high amounts of fibre also leads to an increase in fecal excretion of bile acids and fats (i.e., dietary fibre induced steatorrhea), likely related to an inhibition of pancreatic enzymes [19,20,21,22]. In vitro studies have demonstrated that in the surface of some forms of dietary fibre, bile acids, pancreatic enzymes and minerals can be bound [20,23,24,25]. Schneeman and coworkers studied the effect of purified and unpurified fibre on enzyme activities by testing dietary fibre such as wheat bran, whole alfalfa, rice bran, safflower flour, cellulose acetate, xylan, Solka-Floc and pectin. Chymotrypsin activity was extensively dose-dependently reduced only by Solka-Floc purified fibre. Lipase activity, in turn, was significantly lost with almost all of the purified and non-purified fibre tested, excluding pectin [26]. Furthermore, the addition of pectin, guar gum and wheat bran to the human duodenal juice reduces the activity of amylase and lipase. Pectin and wheat bran also reduced the activity of trypsin, only pectin caused a reduction in phospholipasic activity [24]. However, these studies were conducted in vitro and how these findings can be translated to humans is currently unclear.

Fibre inhibition of pancreatic enzymes has been hypothesised to be due to compounds present in plants, better known as “anti-nutrients” [27]. Gemede A. and Ratta N. defined anti-nutrients as “anti-nutritional factors with negative effects or non-nutritive compounds with positive effects on health” [28]. The negative or positive effects of these compounds depend on the specific clinical context. Anti-nutrients can reduce blood sugar levels and insulin responses to starchy foods and/or reduce cholesterol plasma levels and triglycerides levels, making them potentially useful as an anti-obesity drug [29]. In turn, these anti-nutrient compounds could exacerbate malabsorption in pancreatic insufficiency, negatively affecting the management of some pancreatic disorders. Anyhow, all studies available on anti-nutrients are in vitro and the results must be taken with caution. The effect of these substances should be evaluated in vivo in order to assess their effect in patients with pancreatic disease. Principle food sources of anti-nutrients with inhibitory effects on pancreatic enzyme activity are reported in Table 1. 

Anti-nutrients main effect is to act as pancreatic lipase inhibitors and hence to interfere with macro- and micronutrient absorption. Different natural substances, contained in different officinal plants and vegetables, have been screened for lipase inhibitors activity [30]. Based on their chemical structure, these can be grouped into seven classes: saponins, polyphenols, terpenes, glycosides, alkaloids, carotenoids and polysaccharides [31]. More specifically, the main lipase inhibitors are green tea with epigallocatechin-3-gallate, ginseng with saponin, extracts of peanut (Arachis hypogaea), alkaloids with caffeine, theophylline and theobromine, glycoside in Glycyrrhiza glabra, terpenes in Salvia officinalis, procyanidin fraction of the polyphenol extract [32], as well as soybean extracts [33]. Several anti-nutrients, such as, phytic acid, lectins, tannins, saponins, act also as amylase inhibitors and protease inhibitors [34,35]. 

### 1.2. Pathophysiology of Digestion and Absorption in Pancreatic Diseases

Temporary or long-lasting PEI is a common complication of pancreatic diseases, whose incidence is disease-related. 

Acute pancreatitis (AP) is an inflammatory condition of the pancreas that often involves peri-pancreatic tissues and remote organ systems. The incidence of AP ranges from 5 to 30 cases per 100,000. The severity of the disease varies widely from the most frequent mild forms to severe disease with multi-systemic organ failure, occurring in about 10–20% of cases, that can eventually lead to death [36,37,38]. A recent meta-analysis reported that PEI occurs in about 25% of patients, during follow-up, after an AP episode. Main risk factors are an alcoholic etiology and AP severity with a significant loss of pancreatic parenchyma [39].

Chronic pancreatitis (CP) is a fibro-inflammatory syndrome of the pancreas, characterised by irreversible morphological changes, that typically causes abdominal pain and can eventually result in permanent loss of the pancreatic function [40,41]. The clinical presentation of CP depends on the disease stage: Early phases are characterised more frequently by episodes of abdominal pain, while, as the disease progresses, signs of pancreatic exocrine and endocrine insufficiency develop [42,43]. PEI also occurs in pancreatic surgical resection, as a consequence of loss of parenchyma.

The main clinical consequence of PEI is malnutrition, consequent to maldigestion-driven malabsorption of macro- and micronutrients, like fat-soluble vitamins, minerals and trace elements [44]. An adequate diet is a cornerstone in the management of pancreatic disease that is required to ensure adequate nutritional intake and to limit malabsorption. For this reason, a nutritional screening becomes crucial. According to the European Society of Clinical Nutrition and Metabolism (ESPEN), the diagnosis of malnutrition should be based on either a low body mass index (BMI), or on the combined weight loss associated with reduced BMI (age-specific) or a low fat-free mass index (FFMI), using sex-specific cut-offs [45]. With the loss of pancreatic parenchyma, a status of altered blood glucose level can also occur that is characterised by phases of hyperglycemia induced by the persistent hepatic glucose production, but also by frequent episodes of hypoglycemia because of increased peripheral insulin sensitivity [46,47]. 

The pathogenesis of glucose homeostasis disorders in specific pancreatic disease is heterogeneous, as the terminology used to describe it. The previous term “type 3-c diabetes”, which was used from 2002 to 2014 in the classification of diabetes by the American Diabetes Association (ADA), is now considered old and confusing and has been replaced with the new term “diabetes of the exocrine pancreas” (DEP) [48]. Criteria for the diagnosis of DEP, published in 2016, are as follows: 1. the presence of PEI (confirmed by monoclonal fecal elastase-1 testing or direct function tests); 2. consistent pancreatic abnormalities on imaging (endoscopic ultrasound, MRI, or CT scan) and; 3. absence of related autoimmune markers of type 1 diabetes [49]. DEP is further split into two categories. ‘New onset diabetes after pancreatitis’ (NODAP) that acknowledges the effect of acute or chronic pancreatitis on previously normal glucose homeostasis, excluding patients with diabetes, before and up to 3 months after hospital discharge with pancreatitis; and ‘post-pancreatitis diabetes mellitus’ (PPDM), which describes the presence of diabetes in the setting of acute or chronic pancreatitis irrespective of the timing of diabetes onset [48]. In literature the estimated probability to develop DEP during CP is around 30–40% [50,51]. In addition, about 40% of patients develops transient abnormal glucose metabolism after only a single episode of AP [52,53], with increased risk of developing diabetes compared to the general population [54]. 

It is important to investigate the occurrence of DEP, in order to start the correct therapy and to avoid malnutrition and control the glycemic profile. In particular, the incretin system may play an important role in the metabolic control of DEP, in which the regulation of the beta-cell mass and the physiological incretin secretion are directly dependent on normal exocrine pancreatic function and fat hydrolysis [55]. Pancreatic diseases have been associated with a functional impairment of the incretin system [56,57]. Therefore, the mainstay of DEP is the deep interaction between exocrine and endocrine pancreas [58].

The target of medical nutrition therapy in diabetic patients, according with the ADA, is to preserve optimal metabolic outcome and to prevent and treat complications. People with overall diabetes are encouraged to choose a variety of fibre-containing foods because they reduce the fasting plasma glucose level, through their slowing effect on gastric emptying and small bowel transit. Last but not least, fibre is able to increase insulin sensitivity. However, in DEP, a fibre-rich diet could inhibit enzymatic activity, worsen maldigestion and consequently lead to malabsorption and incretins release. In patients with DEP and concomitant PEI, oral pancreatic enzyme replacement therapy, necessary for adequate fat digestion and nutrients absorption, normalises the impaired glucagon-like peptide 1 secretion, improving glucose tolerance by maintaining incretin secretion [59,60]. 

The objective of formulating an adequate diet for patients with pancreatic disease is precisely to avoid unjustified food restrictions and to correct nutritional deficiencies, minimise weight loss and prevent excessive gland stimulation to counteract the pain. Correct management of nutrition is achieved by a suitable daily energy intake, with about 55–60% of the calories deriving from carbohydrates (in particular complex carbohydrates and grains), 25–30% deriving from lipids and a protein intake of about 0.8–1 g of protein for kilogram of desirable body weight [61]. While experiencing a pancreatic disease, the nutritional recommendations are to have a personalised dietary counseling in order to assess nutrition and clinical status including anthropometric and biochemical parameters, and finally, to research an eventual exocrine and endocrine dysfunction affecting normal digestion and absorption of macro- and micronutrients [62]. A personalised diet should ensure the right intake of calories and correct nutritional deficiencies, if any, and choose tolerable food in order to reduce the worsening of malabsorption-related abdominal symptoms and avoid the risk of insufficient food intake [63].

## 2. Use of Fibre in Pancreatic Diseases

As discussed previously, the effect of a high fibre diet in patients with pancreatic disease is still debated. On the one side, studies have shown its beneficial role in AP on reducing hospital stay and complications [64,65], on the other side, when the disease progresses and PEI occurs, fibre consumption seems to worsen enzyme function and abdominal symptoms (i.e., diarrhea with or without steatorrhea, pain, bloating) [66,67]. In patients with pancreatic disorders, the correct consumption of fibre might then require a patient-targeted prescription, that takes into account the severity of pancreatic disease, the exocrine and the endocrine pancreatic status [62,63]. 

### 2.1. Fibre in Acute Pancreatitis

Historically, the standard therapeutic approach in the management of AP consisted of the reduction of pancreatic exocrine secretion by “pancreatic rest” obtained via stopping oral feeding. This concept has changed since the last decade. In the absence of ileus or vomiting, even in the acute phase, oral feeding can be initiated early (within 24 h) as tolerated, if the pain is decreasing and inflammatory markers are improving [36]. When patients are subjectively hungry, early refeeding with a solid, low fat and low fibre diet may be safe, regardless of resolution of abdominal pain and normalisation of pancreatic enzymes [68,69].

In AP, nutrition and nutritional supplements are important not only in restoring energy balance, but also in preserving gut barrier function and providing important immunomodulatory and antioxidant effects [70]. Intestinal barrier dysfunction is associated with translocation of bacteria, whose inflammatory and toxic products can migrate and cause infection of the necrotic pancreas, and in the worst scenario, can induce systemic inflammatory response syndrome (SIRS). Therefore, conserving the integrity of the gut barrier in the small intestine is one of the main goals in early-phase treatment of severe AP [71,72]. 

Prebiotics are able to regulate the homeostasis of the intestinal barrier and can be effective in the management of patients with AP [73], even if studies that assess the effect of prebiotic in AP are scarce and heterogeneous (Table 2). Olah and colleagues showed a beneficial effect of synbiotics on severe AP in two randomised, double-blind clinical trials (RCT) [64,74]. In the first one, a beneficial effect on AP-associated endotoxemia was reported in 45 patients receiving, with early enteral nutrition (EN), either live or heat-inactivated *Lactobacillus plantarum 299* with oat fibre supplement. Results showed that live probiotics were effective in reducing pancreatic sepsis and the number of surgical interventions [74]. Their next RCT study involved 62 patients who received “Synbiotic 2000”, a composition containing four different lactobacilli preparations plus prebiotics (four bioactive fibres, inulin, beta-glucan, resistant starch and pectin), compared to a control group, which received prebiotic alone [64]. Patients receiving synbiotic therapy showed reduced incidence of SIRS and lower rates of organ failure, supporting the findings that early EN with synbiotics may prevent organ dysfunctions in the late phase of severe AP. Finally, in a randomised, double-blind study, with 30 consecutive severe AP patients, the use of EN with only prebiotic fibre supplementation was found to shorten hospital stay, duration of nutrition therapy and to reduce the acute phase response and overall complications, compared to standard EN therapy [65].

However, the results of these studies concerning the efficacy of prebiotics in AP should be taken with caution since only the study of Karakan and colleagues [65] directly demonstrated it. The other studies showed the efficacy of synbiotics, namely a combination of prebiotic and probiotic, making it not possible to establish the real effect of prebiotic supplementation on acute pancreatitis outcomes.

### 2.2. Fibre in Chronic Pancreatitis

In patients with chronic pancreatitis (CP), frequent low-volume meals and avoidance of foods that are difficult to digest are generally recommended for the progressive appearance of pain [75]. Malabsorption of micro- and macronutrients connected to the insufficient level and/or activity of pancreatic enzymes is the major cause of progressive nutritional and metabolic impairment in these patients [40]. Fat restriction is no longer recommended, since studies on the fate of both endogenous and exogenous enzymes, during small intestinal transit, show that survival of enzyme activity is enhanced by the presence of their respective substrates [76]. This means that survival of lipase activity during intestinal transit requires the presence of dietary triglycerides [77]. 

**Figure 1:**
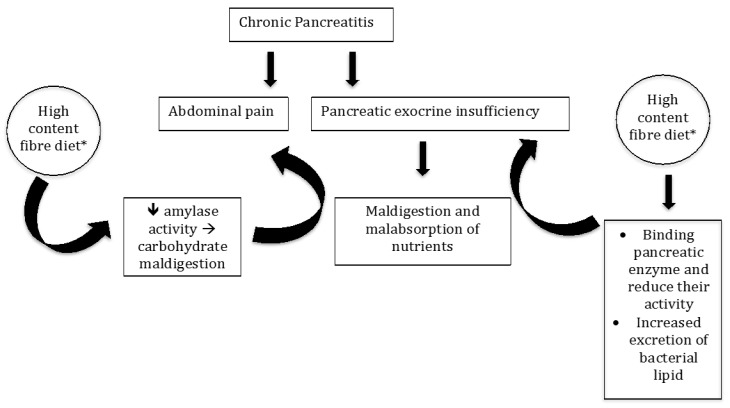
Interaction between high content fibre diet* and pancreatic enzymes in chronic pancreatitis. * High content fibre diet: >25 g/day in women, >38 g/day in men [12].

In patients with PEI, an increase of the fibre content in their diet is associated with a significant rise in the wet fecal weight and fecal fat excretion; the excretion of fecal fat in these patients under a high fibre diet was statistically higher than the excretion of fat in the low fibre diet [19]. The study group was small (only 12 patients) and the amount of fibre intake (80 g/day) was far above the actual high content fibre diet [13]. Of note is that the increase in fecal fat excretion might be due in part to the unabsorbed lipid present in the dietary fibre or to the increased excretion of bacterial lipids. The contribution of bacterial lipid to steatorrhea in patients with pancreatic insufficiency on a high-fibre diet is controversial. First, the fecal flora in human subjects includes a predominant anaerobic bacterial population that has little or no lipids in the cell wall [78,79,80]. Secondly, bacterial excretion in feces is not likely to increase in response to a 1 week high fibre diet [81,82], but decreases after 3 weeks on a high fibre diet [83]. Thus, the increase in fecal fat excretion on high fibre diet ingestion in patients with PEI is most likely due to enhanced fat malabsorption. Indeed, it was calculated in vitro that 1.5 g% of wheat bran, pectin, and cellulose led to maximal reduction of enzymatic activity [19]. Also, the reduction of amylase activity by the dietary fibre may contribute towards carbohydrate maldigestion, and enhance intestinal gas production that could contribute to abdominal pain, frequently detected in these patients. However, it should also be mentioned that symptoms of flatulence and bloating occur even in normal healthy western subjects when the fibre content of their diet is suddenly increased (Figure 1).

Furthermore, the effect of dietary fibre on pancreatic enzymes increases in an acidic environment. This fact is particularly relevant for patients with PEI who often have, during the post-prandial period, an acidic pH (pH < 4.0) in the upper small intestine due to impairment of bicarbonate secretion [66,67,84]. 

Besides maldigestion, patients with CP have recurrent abdominal pain that likely can be triggered by excessive pancreatic secretion stimulation. Evidence suggests that dietary fibre can stimulate the pancreatic gland with an undefined neurohormonal mechanism. Rats fed with a 20% wheat bran supplemented diet for two weeks presented a significant increase in the concentration of lipase, amylase and trypsin in pancreatic tissue [85], however, this was not observed with meals containing cellulose and pectin [86,87]. Similar studies have not been conducted in humans.

In summary, the treatment of pancreatic exocrine deficiency begins with dietary recommendations and pancreatic enzyme supplementation. Anyhow, the amount of fibre should be well thought out in the prescription of a diet in patients with PEI, especially if malnutrition is concomitant. About 80% of patients can be managed by a combination of dietary recommendations, pancreatic enzyme supplements and analgesics, while 10–15% need oral nutritional supplements, 5% need enteral tube feeding and around 1% require parenteral nutrition [75,88,89,90]. 

## 3. Conclusions

The role of fibre intake in the management of patients with pancreatic diseases is still debated and in vivo human studies are lacking. Human studies are hindered by the lack of specific pancreatic “disease indicators” of pancreatic exocrine function that can be helpful in clinical trials, as well as, by the dichotomy of fibre effects between different pancreatic conditions. 

In fact, data from literature shows that in acute pancreatitis, especially in early phase and in mild disease, the use of soluble fibre can be useful to prevent local and systemic complications and to reduce the hospital stay. However, the available studies have been mainly conducted testing the synbiotic effect of concomitant prebiotics and probiotics supplementation. In turn, in chronic pancreatitis, the use of fibre needs to be carefully controlled due to the reported evidence that fibre reduces the effect of pancreatic enzymes, even if the actual recommendations of fibre use or avoidance in the nutritional management of these patients derive almost from in vitro studies. Nevertheless, in these patients, the use of synbiotics [91] improves absorption of micronutrients, even if it does not ameliorate the overall nutritional status. It might be through the modulation of the dysbiosis, a condition recently reported in patients with pancreatic disorders [92].

Translation studies and clinical trials are necessary to better define the link between pancreas and microbiota in humans, but the new evidence of an intricate pancreas–microbiota cross talk in healthy and pathological conditions [92] represents a good target for possible diet interventions. Since, from the current knowledge, probiotics results are useless in acute diseases [93], possible future options could be the use of fibre that greatly impacts microbiota. In this perspective, a topic that warrants immediate attention is to verify the direct role of fibre effects on pancreatic exocrine secretion in vivo in human studies. From the available in vitro data, the choice of the type of fibre to be used requires particular attention. Pure soluble fibre extracts, deprived of anti-nutrients, could be the first choice.

In conclusion, many unanswered questions remain and there is a requirement for continued research into many aspects of the nutritional management of pancreatic disease. At the moment, the mainstay of the use of fibre in the management of pancreatic diseases is represented by the presence or absence of PEI and, according to the current understanding, the use of fibre should then be patient- and disease-targeted, considering specific case-by-case individual clinical conditions.

## Figures and Tables

**Table 1:** Main anti-nutrients acting as inhibitors of pancreatic enzymes and their food source.

Anti-Nutrients	Alimentary Products
Saponins [34]	soybeans
Phytate [27]	grains (rice polished, wheat bran), oilseeds, legumes
Lectins [28]	legumes, oilseeds (soybeans)
Trypsin inhibitors [27]	soybeans, legumes (beans, peas)
Tannins [27]	legumes (beans, peas), cereals (sorghum, millet)
Polyphenols [30]	extracts of citrus fruits, grape seeds, tea (oolong tea), peanut shells, apples

**Table 2:** Effects of pre- and probiotic supplementation in enteral nutrition on acute pancreatitis.

Study	*N* Patients	Pre-Probiotic Group	Control Group	Outcomes
Olah A. (2002) [74]	45	Live *L. plantarum 299* + oat fibre	Inactiveted *L. plantarum 299* + oat fibre	↓ infected pancreatic necrosis (1/22 (4.5%) vs. 7/23 (30.4%) *p* < 0.05) and surgical interventions (1/22 (4.5%) vs. 7/23 (30.4%) *p* < 0.05).
Olah A. (2007) [64]	62	Inulin, beta-glucan, resistant starch, pectin + Lactobacilli	Inulin, beta-glucan, resistant starch, pectin	↓ incidence of SIRS and OF (8/33 (24.2%) vs. 14/29 (48.3%) *p* < 0.05); ↓ overall complications (9/33 (27.3%) vs. 15/29 (51.7%) *p* < 0.05).
Karakan T. (2007) [65]	30	Soluble + Insoluble fibre	Standard enteral solution	↓ hospital stay (10 ± 4 vs. 15 ± 6 *p* < 0.05), overall complications (7/15 (46.6%) vs. 9/15 (60%) *p* < 0.05).

↓: reduction; SIRS: Systemic inflammatory response syndrome; OF: Organ failure.
